# Hyaluronan Fragments Improve Wound Healing on *In Vitro* Cutaneous Model through P2X7 Purinoreceptor Basal Activation: Role of Molecular Weight

**DOI:** 10.1371/journal.pone.0048351

**Published:** 2012-11-16

**Authors:** Kamelia Ghazi, Uriell Deng-Pichon, Jean-Michel Warnet, Patrice Rat

**Affiliations:** Chimie-Toxicologie Analytique et Cellulaire (EA 4463), Université Paris Descartes, Sorbonne Paris Cité, Faculté de Pharmacie, Paris, France; Université de Technologie de Compiègne, France

## Abstract

**Background:**

hyaluronan biopolymer is used in dermatology but the underlying mechanism and the impact of its molecular weight have not yet been investigated in skin wound healing. The aim of our work was to study the role of HA molecular weight in the proliferative phase of wound healing and to understand how this physiological biopolymer acts to promote wound healing on a human keratinocyte in vitro model.

**Methodology and Findings:**

wound healing closure was evaluated using scratch test assay, cell proliferation by counting cell with haemocytometer, expression of CD44 and ZO-1 (protein present in tight junctions specific of epithelia) using flow cytometry, and P2X7 receptor activation on living using a cytoflurometric method. Our study showed that medium hyaluronan fragment (MMW-HA, between 100 and 300 kDa) induced a significant increase in wound closure, increased ZO-1 protein expression and induced a slight activation of P2X7 receptor, contrary to high (between 1000 and 1400 kDa) and low (between 5 and 20 kDa) molecular hyaluronan fragments that had no healing effects. Basal activation of P2X7 receptor is already known to stimulate cell proliferation and this activation in our model plays a pivotal role in MMW-HA-induced wound healing. Indeed, we showed that use of BBG, a specific inhibitor of P2X7 receptor, blocked completely the beneficial effects of MMW-HA on wound healing.

**Conclusion:**

taken together, our results showed for the first time the relationship between P2X7 receptor and hyaluronan in wound healing, and that topical use of MMW-HA (fragment between 100 and 300 kDa) could represent a new therapeutic strategy to promote healing.

## Introduction

Hyaluronan (HA), the major component of the ECM, is the only linear non-sulfated glycosaminoglycane composed of alternating β-1,4-glucuronic acid and β-1,3-N-acetylglucosamin [1]; [2]. HA molecular weight varies from 10^5^ to 10^7^ Da before being progressively degraded into smaller fragments in the ECM [3]. HA was first isolated from the vitreous humour of bovine eyes [4]. It is also present in synovial fluid [5] and is distributed ubiquitously in vertebrate tissues. The major physiological roles of high molecular weight HA in human body are to lubricate articulations and to maintain the cohesion and structure of epithelium. HA performs many pivotal structural and physiological functions in skin repair following injury [3]; [6]. Wound healing consists of 3 processes: inflammation, proliferation and remodelling. During the inflammatory phase of wound healing, hyaluronan accumulates in the wound bed. Its major function is the modulation of inflammatory cell and dermal fibroblast activities, e.g. cellular migration, proinflammatory cytokine synthesis and the phagocytosis of invading microbes [6]. Low molecular weight HA binds to TLR4 to induce inflammatory responses stimulating IL-6, TNFα and IL-1β [7]. Proliferative step is a set of complex biological responses requiring extracellular matrix (ECM) and cytoskeletal remodelling, signal cascades, and gene regulation to induce fibroblast and keratinocyte migration and proliferation. The levels of HA synthesized by both fibroblasts and keratinocytes are elevated during re-epithelialization. HA binds to cell via several receptors: CD44, RHAMM and ICAM. CD44 is recognized as the major receptor of HA [8]; [9]. The interaction of HA with CD44 induces many physiological events, such as cell migration and proliferation [10]; [11]. CD44 is localised in lipid raft domains which are plasma membrane domains that contain high levels of cholesterol and sphingolipids. Proteins such as receptors involved in cell signalling are enriched in lipid rafts [12]. P2X7 receptor, contained in lipid rafts, is a purinergic receptor; it was first cloned from rat brain [13] and, subsequently, has been found to be expressed in microglia, neurons, and astrocytes [14]–[16]. P2X7 receptor was activated by extracellular ATP and its analogues [17]; [18] and blocked specifically by Brillant Blue G (BBG) [19]–[21]. Our laboratory showed that HA of molecular weight superior to 100 kDa modulates the activation of P2X7 receptor via binding to CD44 in ophthalmic cells [22]; [23]. P2X7 receptor has been reported to play a role in cell-to-cell contact [19] and cell proliferation [24]. Moreover P2X7 receptor activation seems to be associated to corneal wound healing [25], Mankus *et al* showed that P2X7 receptor activation enhanced cell migration [26].

**Table 1:** Molecular weights of hyaluronan used.

Nomenclature	Molecular weight polydispersity	Average molecular weight
High Molecular Weight Hyaluronan (HMW-HA)	1000<MW<1400 kDa	1090 kDa
Medium Molecular Weight Hyaluronan (MMW-HA)	100<MW<300 kDa	166 kDa
Low Molecular Weight Hyaluronan (LMW-HA)	5<MW<20 kDa	20 kDa

The aim of our work was to study the role of HA molecular weight in the proliferative phase of wound healing and understand how this physiological biopolymer acts to promote wound healing on a human keratinocyte in vitro model. We evaluated wound healing closure, cell proliferation, expression of CD44 and ZO-1 (protein present in tight junctions specific of epithelia), and P2X7 receptor activation after incubation of cells with a native HA and two different fragments of HA.

**Figure 1:**
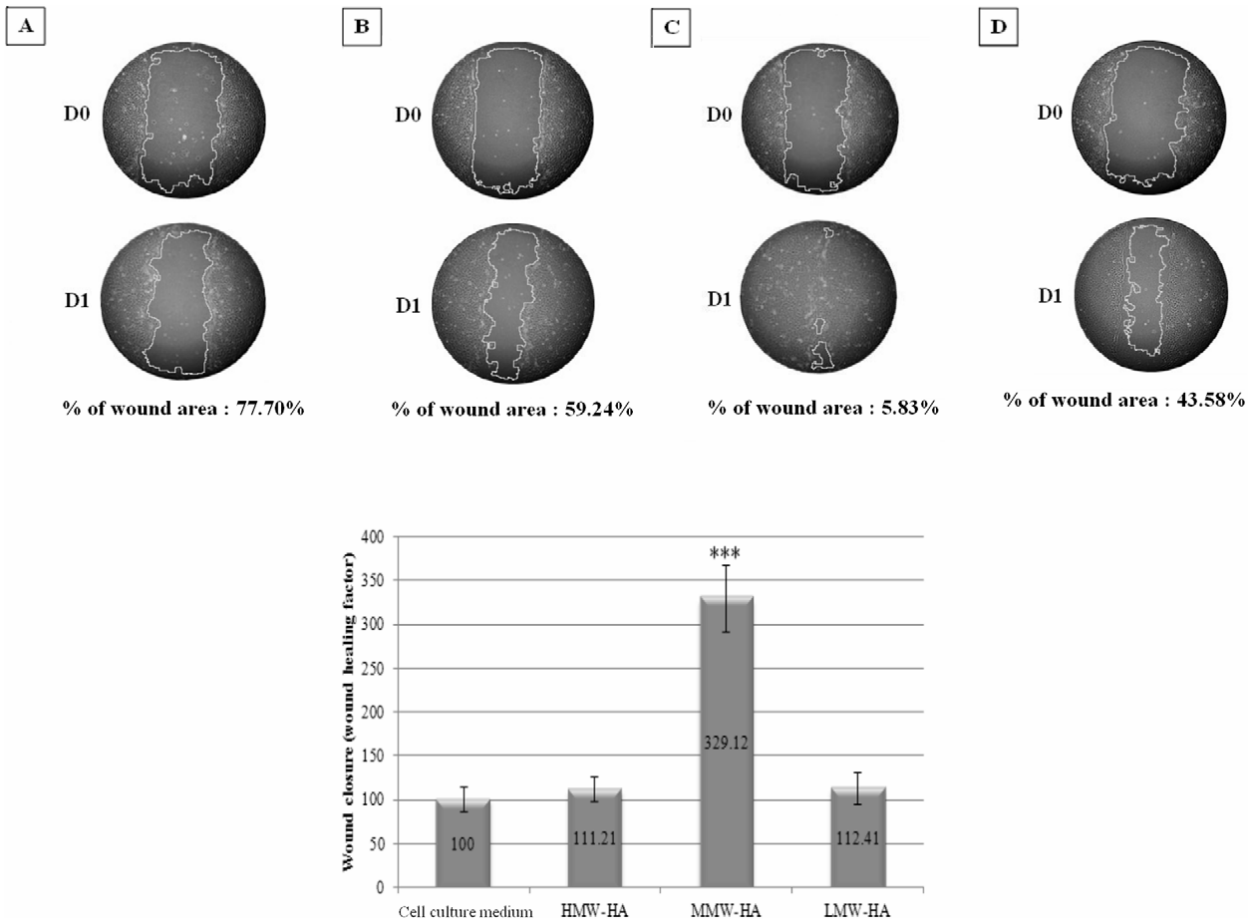
Scratch wound assay. Monolayer was wounded by manual scratch with a pipette tip. Solutions containing different HA at 0.2% in culture medium with 2.5% of FCS were distributed at Day 0 and cultures were kept at 37°C for 24 hours (Day1). A: control, B: HMW-HA, C: MMW-HA, D: LMW-HA. Histogram above represents width of the wound measured for each wound at D0 and D1. Results are expressed as a ratio of D1 on D0. **Wound healing area**: Results are expressed as a ratio of D1 on D0 after image analysis; % of wound area represents the ration of wound area at D1/wound area at D0. We show that only MMW-HA induced a significant wound healing compared to control and other HA (***: p<0.001 compared to culture medium, n = 3). The wound healing factor is three times higher compared to control.

## Results

### Scratch Wound Assay

To study wound healing, we use three different HA molecular weight (table 1). Results showed that, only MMW-HA induced a statistically significant effect on wound healing: the wound healing factor is three times higher compared to control (Figure 1). HMW-HA and LMW-HA had no effect on wound healing compared to control. % of wound area was 77.70%, 59.24%, 5.83% and 43.58% respectively for culture medium, HMW-HA, MMW-HA and LMW-HA.

**Figure 2:**
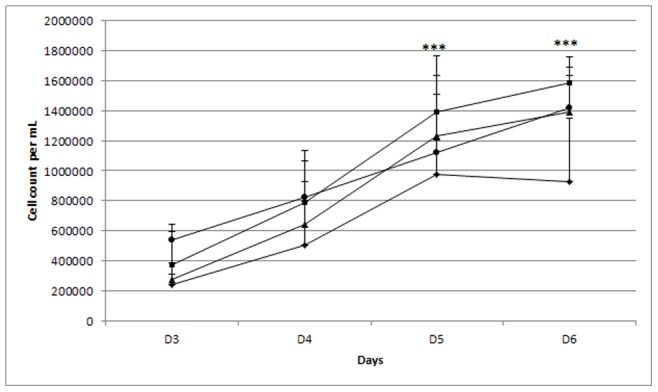
Cell proliferation. Cells were cultured in medium with 2.5% FCS containing HA HMW-HA or MMW-HA or LMW-HA at 0.2%. Proliferation was analyzed by counting cells during 6 days. circle: cell culture medium, triangle: HMW-HA, square: MMW-HA, lozenge: LMW-HA. Our results show that from day 5 only MMW-HA induced a significative increase on cell proliferation compared to control, HMW-HA and LMW-HA have no effect on cell proliferation (***: p<0.001 compared to culture medium, n = 3).

**Figure 3:**
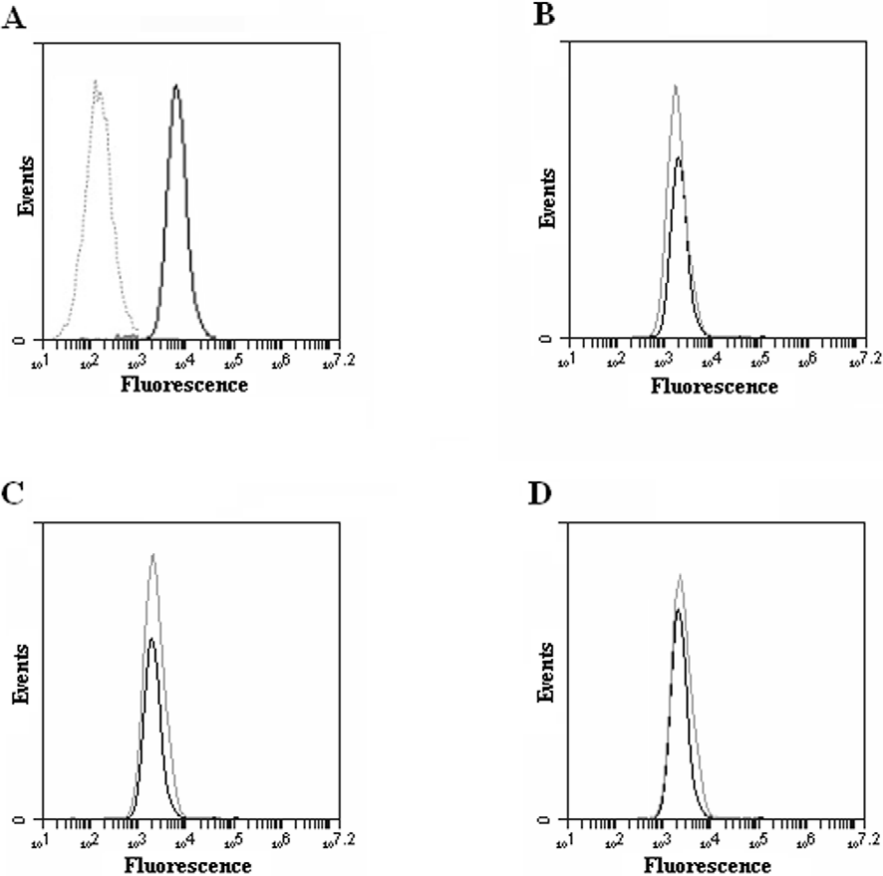
ZO-1 protein expression. ZO-1 expression was evaluated using flow cytometry after incubation with HMW-HA or MMW-HA or LMW-HA at 0.2% for 24 hours. Results showed a strong increase in ZO-1 expression after MMW-HA incubation. A: cell culture medium, B: HMW-HA, C: MMW-HA, D: LMW-HA (n = 3). Data show that MMW-HA increase the expression of ZO-1 protein compared to control.

### Cell Proliferation

The results show that from Day 5, MMW-HA increased significantly cell proliferation compared to control. High and low molecular weight hyaluronan had no significant effect on cell proliferation (Figure 2).

**Figure 4:**
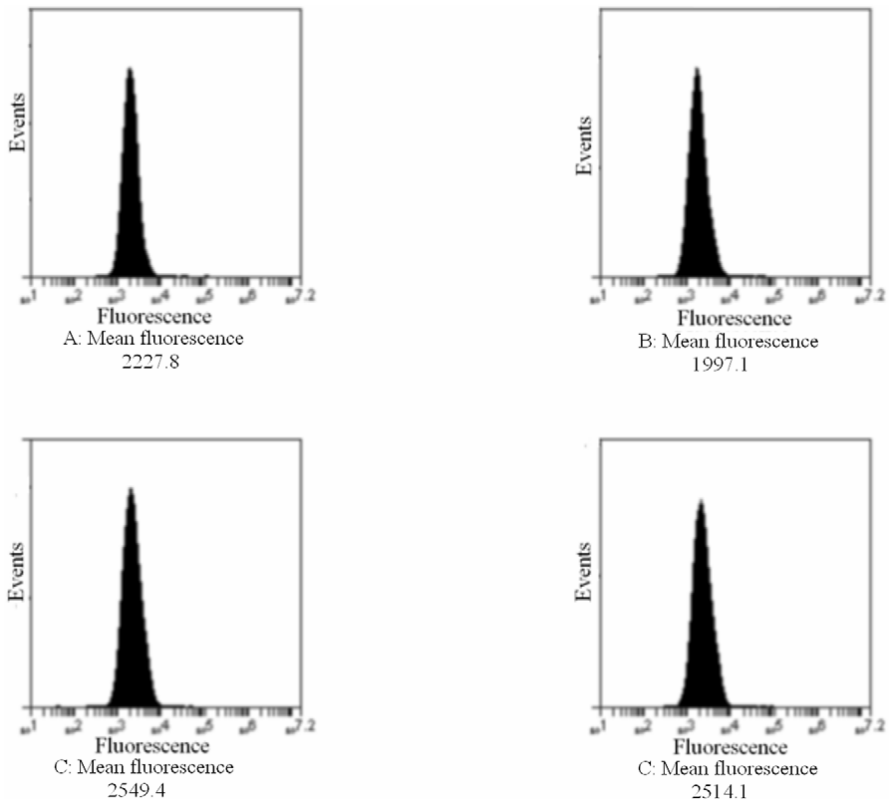
CD44 protein expression. CD44 expression was evaluated using flow cytometry after incubation with HMW-HA or MMW-HA or LMW-HA at 0.2% for 24 hours. Results showed a very slight effect on CD44 expression level after MMW-HA incubation. A:control, B: HMW-HA, C: MMW-HA, D: LMW-HA (n = 3). Results show that CD44 expression is not modified with HA regardless the molecular weight.

### Tight Junction: ZO-1 Expression

As shown in figure 3, ZO-1 expression was strongly increased when the cells were incubated with MMW-HA (mean fluorescence = 36418.6) compared to control (mean fluorescence, = 17951.3). HMW-HA and LMW-HA had no effect on ZO-1 expression compared to control.

**Figure 5:**
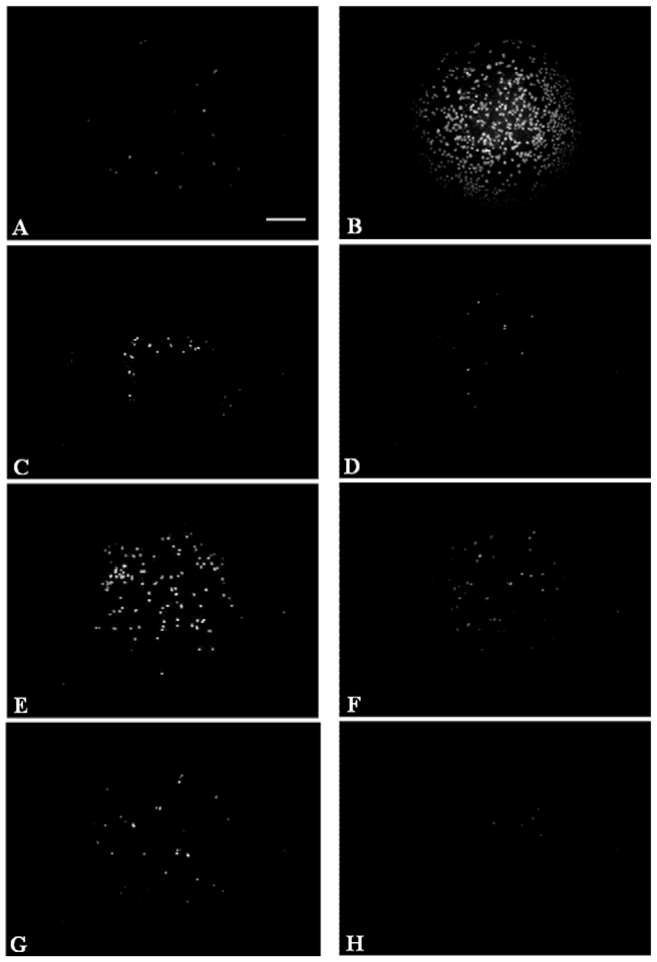
P2X7 receptor activation. Cells were pre-incubated or not with a specific inhibitor of P2X7 (BBG) at 20 µM for 20 minutes before incubation with HMW-HA or MMW-HA or LMW-HA at 0.2% for 24-hours. P2X7 activation was evaluated by YO-PRO-1 dye uptake. A: cell culture medium, B: positive control, C: HMW-HA without specific inhibitor of P2X7 (BBG), D: HMW-HA with specific inhibitor of P2X7 (BBG), E: MMW-HA without specific inhibitor of P2X7 (BBG), F: MMW-HA with specific inhibitor of P2X7 (BBG), G: FMW-HA without specific inhibitor of P2X7 (BBG), H: LMW-HA with specific inhibitor of P2X7 (BBG). Incubation with MMW-HA induces slight increase in P2X7 activation compared to control and other HA (n = 18). Moreover preincubation of cell with specific inhibitor of P2X7 (BBG) inhibits MMW-HA effect on P2X7 receptor.

**Figure 6:**
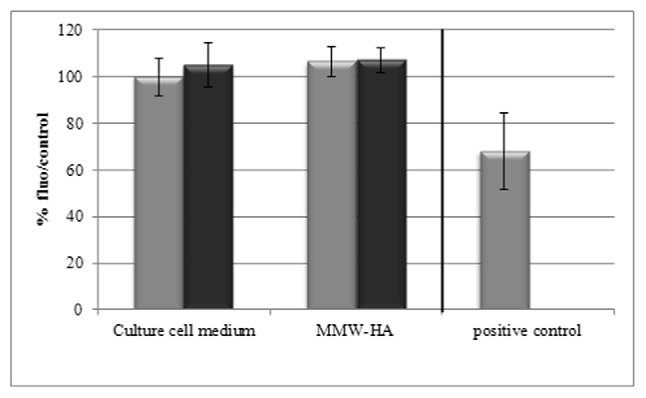
Cell viability. Cells were preincubated or not with specific inhibitor of P2X7 (BBG) at 20 µM for 20 minutes before incubation with MMW-HA at 0.2% for 24-hours. Cell viability was evaluated by neutral red test after incubation with MMW-HA. Grey: without specific inhibitor of P2X7 (BBG), black: with specific inhibitor of P2X7 (BBG). Slight activation of P2X7 we observe with MMW-HA has no effect on cell viability (n = 18).

### Hyaluronan Receptor: CD44 Expression

As shown in figure 4, the three HA had no effect on CD44 expression compared to control.

**Figure 7:**
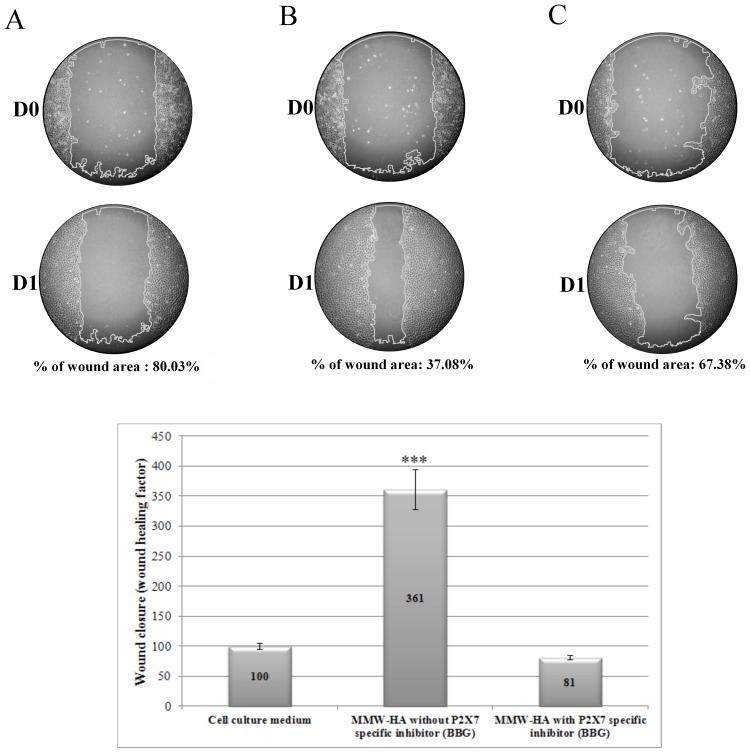
Scratch wound assay. Monolayer was wounded by manual scratch with a pipette tip and cells were preincubated with BBG at 20 µM for 20minutes before HA incubation. Different solutions containing different HA at 0.2% in culture medium with 2.5% of FCS are distributed (Day0) and culture was kept at 37°C for 24 hours (Day1). A: control without specific inhibitor of P2X7 (BBG), B: MMW-HA with without specific inhibitor of P2X7 (BBG), C: MMW-HA with without specific inhibitor of P2X7 (BBG). % of wound area represents the ration of wound area at D1/wound area at D0. We show that cell preincubation with BBG blocks beneficial effect of MMH-HA on wound healing (***: p<0.001 compared to culture medium, n = 3).

### P2X7 Receptor Activation

In cells where P2X7 receptor is activated, we can observe a green fluorescent signal. As shown in figure 5, incubation of cells with MMW-HA induced a slight activation of P2X7 receptor compared to control and this activation was inhibited by BBG, a specific inhibitor of P2X7. HMW-HA and LMW-HA had no effect on P2X7 receptor activation compared to control.

### Cell Viability

HMW-HA, MMW-HA and LMW-HA didn’t induce any decrease in cell viability (figure 6).

### P2X7 Activation and Wound Healing

To know if P2X7 receptor plays a role in wound healing induced by MMW-HA, cells were incubated with BBG at 20 µM for 20 minutes before MMW-HA incubation. Results showed that preincubation with specific inhibitor of P2X7 (BBG) blocked the beneficial effects of MMW-HA on wound healing (figure 7). % of wound area was 80.03%, 37.08% and 67.38% respectively for culture medium, MMW-HA without BBG and MMW-HA with BBG.

## Discussion

In clinics, some sticking plasters are composed of HA for its hygroscopic properties (hydrated HA can contain up to 1000-fold more water than its own weight). High molecular weight HA has the ability to absorb water and then it keeps the wound in a moist environment favourable to healing. Nevertheless, HA has also biological properties that could explain its role in wound healing. We found that from a physiobiological point of view, MMW-HA induced a much better healing rate than LMW-HA and HMW-HA. Indeed, we demonstrate that native HA, in spite of its essential hygroscopic role, is not the best HA to promote wound healing, and smaller molecular weight (300 kDa) HA seem to play an important biological role in wound healing. Studies have shown that HA modulate cell proliferation [10]; [27], depending on the molecular weight. Hyaluronidases, that are responsible for the degradation of hyaluronic acid, stimulate cell proliferation [28] leading to the conclusion that high molecular weight HA must be degraded to induce cell proliferation. The importance of HA degradation for cell proliferation was confirmed by a recent study showing that high molecular weight HA decreases astrocytes proliferation, but its degradation into smaller fragments induces astrocytes proliferation and activation [29]. We observed that HMW-HA didn’t induce cell proliferation, showing that hyaluronidases may not be present in our cell model. On the contrary, MMW-HA induced cell proliferation compared to control. LMW-HA had no effect on cell proliferation but this was not surprising since low molecular weight HA is pro-inflammatory [7]; [30]. So, the use of this HA could potentiate inflammation in wound leading to tissue damage. Considering these data, we focused our attention on MMW-HA.

Cell-cell junction/adhesion is an important factor for wound healing. Tight junctions play a central role in close cell-cell adhesion in simple epithelia and endothelia, connecting neighbouring cells in a controlled manner. It is composed of transmembrane proteins including claudin family proteins, occludin like proteins, junctional adhesion molecules (JAMs) and plaque proteins such as ZO-1 [31]. Besides, it plays a crucial role in the formation of the epidermal diffusion barrier. The expression of the tight junction proteins ZO-1 has recently been studied in a human skin organ culture model [32], showing ZO-1 expression early during wound healing. In our model, incubation of cells with MMW-HA induced an increase in ZO-1 expression. Not only MMW-HA stimulated cell proliferation and wound healing, but it also increased the expression of tight junctions, showing that the rebuilt epithelium monolayer possessed functional properties.

To understand how MMW-HA promotes wound closure and cell proliferation, we studied CD44 expression and P2X7 receptor activation. The results we obtained show that incubation of cells with MMW-HA has no significative effect on CD44 receptor expression (major receptor of HA). This indicates that the healing we observed with MMW-HA is not linked to the modulation of CD44 receptor expression, but probably due to other cell signalling pathway. Recently, several studies have highlighted the possibility that HA binding to CD44 induces its redistribution into lipid rafts, with multiple signal molecules being recruited and assembled to facilitate the transduction of signals [33]; [34]. Among the receptors present in lipid raft, there is the P2X7 purinergic receptor. First, P2X7 receptor was identified as a cytolytic receptor [13], but it was later shown that basal activation of P2X7 increased mitochondrial calcium and promoted cell proliferation [35]; [36]. P2X7 receptor activation has a dual role: cell proliferation after basal activation and cytolysis after high activation. The role of P2X7 receptor activation in wound healing was first highlighted in cornea [25]. The same authors showed that in P2X7−/− mice, there was a downward trend in the rate of epithelial wound repair [37]. Our results show that MMW-HA induced a slight activation of P2X7, and this slight activation was not accompanied with a loss of cell viability. This basal P2X7 activation plays a pivotal role in MMW-HA-induced wound healing since BBG, a specific inhibitor of P2X7 receptor activation, inhibited wound closure promoted by MMW-HA. These results showed that when P2X7 receptor is blocked, MMW-HA lost its healing properties. According to our results, MMW-HA (166 kDa) seems to be the best healing agent compared to LMW-HA and HLW-HA. Besides, HA with molecular weight inferior to 200 kDa has been shown to stimulate β-defensin-2 on human skin cells [38]. β-defensin-2, a peptide produced by different epithelial cells, exerts a strong antimicrobial activity against Gram-negative bacteria and Candida albicans, together with a good bacteriostatic activity against Gram-positive bacteria [39]. MMW-HA combines proliferative stimulating effects and indirect antimicrobial activity. These both properties confer to MMW-HA an interesting role in wound healing.

**Figure 8:**
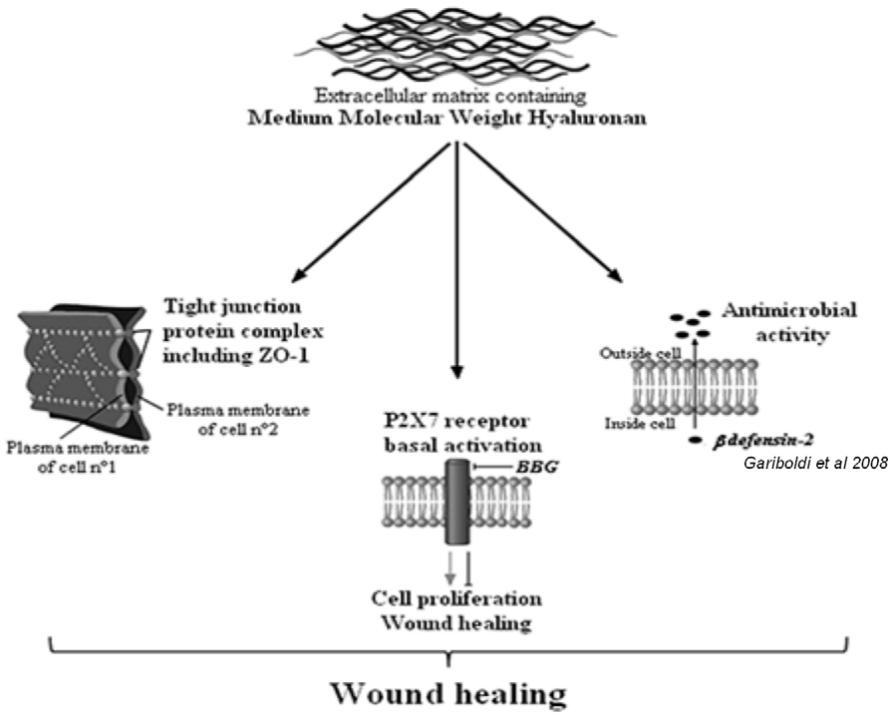
Summary diagram. MMW-HA induces wound healing via P2X7 basal activation.

Our study allowed us to better understand the mechanism by which hyaluronan acts in wound healing (see above summary diagram, figure 8).

We demonstrated that native HA is not the best biopolymer to stimulate wound healing and cell proliferation, but rather HA at 166 kDa represents an interesting therapeutic strategy as it promotes *in vitro* the rebuilt of a functional epithelium monolayer. We highlighted for the first time the relationship between HA at 166 kDa and P2X7 receptor basal activation in wound healing. Together, these results show that topical use of medium molecular weight hyaluronan in sticking plasters should be taken into consideration to promote tissue repair and wound healing.

## Materials and Methods

### Reagents

Chemicals, cell culture reagents and fluorescent dyes were purchased from Sigma-Aldrich (Saint-Quentin-Fallavier, France), Eurobio (Les Ulis, France) and Invitrogen (Cergy Pontoise, France), respectively. Different HA were obtained by biotechnology processes (Laboratoire Soliance, Pomacle, France): one native HA (HMW-HA) around 1 MDa, and two smaller fragments, one medium molecular weight HA (MMW-HA) around 150 kDa and one low moleculear weight HA (LMW-HA) around 15 kDa. Molecular weights were calculated according to European Pharmacopoeia guidelines 6.3. Hyaluronan was used at 0.2%; this concentration was tested according our previous study [23] which are showed that hyaluronan at this concentration had an effect on corneal cells.

### Experimental Procedures

#### Cell culture

HaCat cells (human skin keratinocytes, [40]) were cultured under standard conditions (moist atmosphere of 5% CO_2_ at 37°C) in Dulbecco’s minimum essential medium (DMEM) supplemented with 10% foetal calf serum (FCS), 2 mM L-glutamine, 50 IU/ml penicillin, and 50 IU/ml streptomycin. The medium was changed every three days. Confluent cultures were removed by trypsin incubation, and then cells were counted. They were seeded in culture microplates at a density of 250,000 cells per mL.

Cells were pre-incubated or not with a specific inhibitor of P2X7 (brilliant Blue G- BBG) at 20 µM for 20 minutes before incubation with HMW-HA or MMW-HA or LMW-HA at 0.2% for 24-hours.

#### Scratch wound assay

According to Buonomo’s method [41]–[43], the cells were seeded into 6-well culture microplates, and culture was kept at 37°C for 72 hours. The medium culture was removed and the cells were rinsed with phosphate buffer saline (PBS) and incubated with culture medium without FCS during 24 hours. After 24 hours, medium was removed and the cells were washed and wounded by manual scratch with a pipette tip. The cells were rinsed with PBS and pictures of the wounds were taken using Nikon Coolpix camera. Solutions containing different HA at 0.2% in culture medium with 2.5% of FCS were distributed. Cultures were kept at 37°C for 24 hours. At D1 (+24h), medium was removed and the cells were washed before taking pictures of each wound. % of wound area was measured using Aphelion Dev image processing and analysis software developed by ADCIS S.A.

#### Cell proliferation

Cells were seeded with different HA at 0.2% in culture medium at 80,000 cells per mL into 48-well culture microplates. Each day, the cells were removed with trypsin and counted for 6 days with haemocytometer.

#### Tight junction: ZO-1 expression

ZO-1 expression was evaluated using flow cytometry [44]. Cells were seeded into 6-well culture microplates, culture were kept at 37°C for 24 hours. The cells were incubated with different HA at 0.2% in medium with 2.5% of FCS for 24 hours. After 24 hours, medium was removed and the cells were washed and removed by trypsin. The cells were fixed with 1% paraformaldehyde in PBS. The cells were washed with PBS, and incubated for 30 minutes at 4°C with ZO-1 polyclonal antibodies (ZYMED Laboratories) diluted to 1/100. After washes in cold PBS supplemented with 0.5% of bovine serum albumin (BSA), the cells were incubated for 30 minutes at 4°C with a goat anti-rabbit immunoglobulin-Alexa Fluor®488 diluted to 1/2000. After wash in cold PBS supplemented with 0.5% of BSA, the cells were analysed by C6 Flow Cytometer® (Accuri Cytometers, St. Ives, UK).

#### Hyaluronan receptor: CD44 receptor expression

Flow cytometry were used to evaluate CD44 expression [45]. According to Pauloin’s method [23], cells were seeded into 6-well culture microplates, culture were kept at 37°C for 24 hours. The cells were incubated with different HA at 0.2% in culture medium with 2.5% of FCS. After 24 hours, culture medium was removed and the cells were washed and removed by trypsin. Culture cells were incubated for 45 minutes at 4°C with CD44 monoclonal antibodies (Sigma-Aldrich) diluted to 1/500. After three washes in cold PBS supplemented with 0.5% of BSA, the cells were incubated 30 minutes at 4°C with a goat anti-mouse immunoglobulin-FITC (Dakocytomation, Glostrup, Denmark) diluted to 1/2000. After three washes in cold PBS supplemented with 0.5% of BSA, the cells were fixed with 1% paraformaldehyde in PBS. The cells were analysed by C6 Flow Cytometer® (Accuri Cytometers, St. Ives, UK).

#### P2X7 receptor activation: YO-PRO-1 test

YO-PRO-1, a DNA probe [46], enters cells after P2X7 receptor activation [13]. For this test, cells were seeded into 96-well culture microplates and kept at 37°C for 24 hours. The cells were incubated with different HA at 0.2% in culture medium with 2.5% of FCS. After 24 hours, culture medium was removed and the cells were washed with PBS. According to our laboratory’s protocol [47]; [48], 2 µM YO-PRO-1 solution was distributed in the microplate. After 10 minutes at room temperature in the dark, pictures of cells were taken using a Nikon Coolpix camera connected to a fluorescence microscope Leïca DMIRB.

#### Cell viability

Neutral red (Fluka, Buchs, Switzerland) uptake assay is a cell viability assay, based on the ability of viable cells to incorporate neutral red [49], which is a weak cationic dye that readily penetrates cell membranes by non-ionic diffusion, accumulating in lysosomes, where it binds with anionic sites in the lysosomal matrix. Lysosomal membrane integrity is closely correlated with cell viability and it is evaluated with neutral red fluorescence. Cells were seeded into 96-well culture microplates, culture were kept at 37°C for 24 hours. The cells were incubated with different HA at 0.2% in culture medium with 2.5% of FCS. After 24 hours, culture medium was removed and the cells were washed. Neutral red solution (50 µg/ml) was added to living cells. After a 3-hour incubation time at 37°C, the cells were washed with PBS to remove any remaining unincorporated dye. The dye was then released from the cells using lysis solution (1% acetic acid, 50% ethanol and 49% pure water). The plate was agitated on a microplate shaker for 20 minutes and the fluorescence signal was scanned (λexc = 540 nm; λem = 600 nm) [50], using a cytofluorometer (Safire®, Tecan™, France) which allows fluorometric detection from 280 to 870 nm with high sensitivity (pg-fg/ml) and specificity. This technique allows the use of fluorescent probes on living cells and detects the fluorescent signal directly in the microplate in less than 1 minute (for a 96-well plate).

#### Statistical analysis

Each test was performed in triplicate. The fluorescent measurement was expressed as relative fluorescence units or as fluorescence percentage of the control, and statistical analysis were performed using one-way ANOVA followed by Dunnett’s test (α risk = 0.05). Error bars on graphs represent standard deviation. The significance is compared to control (culture medium). Statistical analysis was performed using Sigma Stat 2.0 software (Chicago, IL, USA).
